# Posters

**DOI:** 10.1038/sj.bjc.6601936

**Published:** 2004-06-22


### EVIDENCE FOR THE ASSOCIATION OF THE 4Q32-34 LOCUS AND PANCREATIC CANCER IN EUROPEAN FPC FAMILIES

J Earl [1], L Yan [1], C McFaul [1], R Kress [2], J Leslie [1], H Rieder [2], M Sina-Frey [2], S Hahn [3], D Bartsch [2], W Greenhalf [1], J Neoptolemos [1]
[1] The University of Liverpool, Liverpool, United Kingdom, [2] Philipps-University, Marburg, Germany, [3] University of Bochum, Bochum, Germany

**Introduction:** Familial Pancreatic Cancer (FPC) is a rare autosomal dominant disease, the causative gene of which is unknown. However, the locus 4q32-34 has been implicated in one large US Kindred. This locus contains genes involved in DNA repair (HMGB2), apoptosis (PPID) and cell cycle control. The European registry of Hereditary Pancreatitis and Familial Pancreatic Cancer (EUROPAC) in collaboration with the FaPaCa group in Germany has recruited over 150 families with pancreatic cancer.
**Aim:** Is the 4q32-34 locus associated with FPC families.
**Methods:** The 4q32-34 locus has been haplotyped in affected and unaffected individuals in 41 EUROPAC and FaPaCa families that fulfil our criteria for FPC. The locus was haplotyped with 9 mapping pairs covering an area of 15cM. Alleles are defined on the basis of size and haplotypes assigned to each individual. High Mobility Group Box 2 (HMGB2) and cyclophillin D (PPID) genes have been sequenced in 10 different FPC families.
**Results:** We have excluded linkage to the 4q32-34 locus in 7 families. Of the remaining 34 linkage cannot be excluded due to either the lack of biological samples or that the disease status of individuals cannot be confirmed as they are considered too young to develop the disease or be classed as unaffected. A total of 55 haplotypes have been identified as possibly being linked to the disease and a further 75 haplotypes have been ruled out. Potential disease causing mutations or nucleotide polymorhisms have not been identified by DNA sequencing of HMGB2 and PPID genes in FPC patients so far.
**Conclusions:** Linkage to the 4q32-34 locus has been excluded in 7/41 families analysed, LOD score analysis will enable us to determine the likelihood of this locus being linked in all of our families. EUROPAC and FaPaCa continue to recruit new families in order to continue the study of this locus and to identify other potential loci.

### P32

### THE CALCIUM BINDING PROTEIN S100A6 (CALCYCLIN) AS A PROGNOSTIC INDICATOR IN PANCREATIC CANCER

D Vimalachandran [1], R Watson [1], A Shekouh [1], C Thompson [1], F Campbell [2], W Prime [2], T Crngorac-Jurcevic [4], N Lemoine [4], J Neoptolemos [1], E Costello [1]
[1] Department of Surgery, University of Liverpool, Liverpool, United Kingdom, [2] Department of Pathology, Royal Liverpool University Hospital, Liverpool, United Kingdom, [3] Department of Human Anatomy and Cell Biology, Liverpool, United Kingdom, [4] Cancer Research UK Molecular Oncology Unit, London, United Kingdom

INTRODUCTION: Pancreatic cancer is a devastating disease, responsible for approximately 40,000 deaths in Europe each year. Using both RNA expression analyses and proteomics based studies we have previously demonstrated increased expression of the calcium binding protein S100A6 in pancreatic cancer. The aim of this study was to determine the significance of S100A6 expression as a prognostic indicator in comparison to other clinicopathologic parameters.
METHODS: Immunohistochemical analysis was performed on a tissue micro-array of sixty pancreatic cancer tissue specimens. The intensity of staining [negative (undetectable), weak, intermediate, strong] was scored along with the proportion of cells staining. For the purposes of analysis, immunoreactivity (expression) was categorised into two groups; undetectable or weak ("low expression") and intermediate or strong ("high expression"). Univariate and multivariate survival analysis was then performed using S100A6 expression and other commonly used clinicopathologic parameters.
RESULTS: High S100A6 expression was observed in 40(80%) of 60 patients. Kaplan-Meier analysis revealed a significantly decreased survival time in high S100A6 expressing cases (p=0.02) when compared with low expression. Nodal metastases (p=0.05) and resection margin involvement (p=0.04) were also significantly associated with survival. Multivariate analysis with a Cox proportional hazards model using S100 expression, nodal status and resection margin status revealed S100A6 expression to be a significant independent prognostic indicator (p=0.008).
CONCLUSIONS: Our findings suggest S100A6 may be at least as powerful a determinant of survival as existing prognostic indicators. The exact role that S100A6 plays in pancreatic adenocarcinoma is at present unknown. The findings of this study, and the implication that S100A6 may play a significant role in pancreatic tumour progression, clearly merit further investigation.

### P33

### IN DESIGNING CLINICAL TRIALS CAN WE PREDICT SURVIVAL OUTCOME FOR OUR PATIENTS WITH RENAL CELL CARCINOMA FOLLOWING NEPHRECTOMY?

GWA Lamb [1], DC McMillan [2], EJ Bromwich [1], PA Vasey [3], M Aitchison [1]
[1] Department Of Urology, Gartnavel General Hopspital, Glasgow, United Kingdom, [2] Department Of Surgery, Glasgow Royal Infirmary, Glasgow, United Kingdom, [3] Beatson Oncology Centre, Western Infirmary, Glasgow, United Kingdom

Introduction: Prognostic algorithm models are of particular value in the design of clinical trials. The UCLA Integrated Staging System (UISS) and The Mayo Clinic Ssign score, are two predictive models, based on American data [*J Clin. Oncol* 2002; 20(23): 4559-4566 / *J Urol* 2002; 168(6): 2395-2400] for stratifying renal cell carcinoma (RCC) patients into prognostic groups following nephrectomy. To date there is no predictive algorithm designed on a UK population base. We evaluated each model to determine if they are applicable to a UK population.
Patients and Methods: 145 patients (95 male, 50 female) with clear cell tumours and for whom all the data required to calculate a predictive score for both models was available, were identified retrospectively from the 502 patients in our renal cancer database. 29 were node positive or had had cytoreductive nephrectomy. The remaining 116 had had potentially curative surgery. Cox regression analysis was used to define hazard ratios (HR) and survival.
Results: Twenty nine disease specific deaths were recorded (13 from potentially curative group, 16 from advanced group). Median follow up was 39 months. Both the Ssign score (HR 1.36, 95%CI 1.12-1.64, p=0.002) and UISS (HR 1.87, 95%CI 1.44-2.42, p<0.001) were highly significant at predicting survival outcome following nephrectomy. Only the UISS score remained significant (HR 3.84, 95%CI 1.39-10.58, p<0.009) when patients with metastatic disease were excluded from analysis.
Conclusion: These models predict outcome following nephrectomy when applied to a UK population. The Ssign score was a good predictor of overall mortality however relied on patients with metastases at the time of surgery to maintain significance. Only the UISS system significantly predicted outcome in both localised and metastatic disease and is the model we recommend for use in the U.K. when designing clinical trials.

### P34

### A COMBINED ONCOLOGY/ SURGICAL RENAL CANCER CLINIC PROVIDES A COMPREHENSIVE CANCER SERVICE AND INCREASES RECRUITMENT FOR CLINICAL TRIALS

GWA Lamb [1], PA Vasey [2], EJ Bromwich [1], M Aitchison [1]
[1] Department Of Urology, Gartnavel General Hospital, Glasgow, United Kingdom, [2] Beatson Oncology Centre, Western Infirmary, Glasgow, United Kingdom

Introduction: Since 1999 our regional referral centre has provided a combined oncology/ surgical clinic for patients diagnosed with metastatic renal cancer (RCC). RCC is a rare tumour accounting for 2% of all adult malignancies. Survival benefit has been demonstrated combining treatments of immunotherapy, cytoreductive nephrectomy, excision of local recurrence and solitary metastasectomy. The provision of a combined service allows for rapid assessment and constructive management planning using these various treatment modalities and higher levels of recruitment for clinical trials.
Materials and Methods: The clinic is lead by a consultant oncologist and a consultant urological surgeon. A cardiothoracic and a general surgeon are available for consultation at short notice. A dedicated clinical research nurse based in the clinical research unit running several clinical trials in renal cancer simultaneously supports the clinic.
Results: Between1999 and 2003 386 new patients have been seen at the combined clinic. 26 patients underwent cytoreductive nephrectomy, 24 metastasectomy and 8 excision of local recurrence. A database of 526 patients with RCC has been established. Trials include; EORTC U70 (Adjuvant RCC)-21 patients entered, UK total entry 126 (23 centres). U79 MRC RE04 (Advanced RCC)-29 patients entered, UK total entry 311 (28 centres). U80 ICRF (Advanced RCC)- 4 patients entered.
Discussion: On their initial visit patients are assessed for trial eligibility and surgery by both consultants within the primary team. Follow up is at the same clinic allowing surgical and oncological complications to be dealt with by the appropriate specialist. More patients attending our institution are proceeding to surgical intervention in the course of their treatment. Our single institution accounts for 16.7% and 9.3% total UK recruitment of the U70 adjuvant and U79 trials respectively. A combined oncology/ surgical renal cancer clinic provides patients with a comprehensive cancer service on a single visit which benefits from increased recruitment for clinical trials.

© 2004 Cancer Research UK

### P35

### A RANDOMISED PHASE II STUDY OF INTERFERON-A ALONE OR IN COMBINATION WITH THALIDOMIDE IN METASTATIC RENAL CELL CARCINOMA

S.R Muthuramalingam [1], S Madhusudan [1], P.A Vasey [2], P Patel [3], K Christodolus [1], A Protheroe [1], A.L Harris [1]
[1] United Kingdom, CRUK Medical Oncology Unit, Churchill Hospital, Oxford, OX3 7LJ, [2] United Kingdom, CRC Department of Medical Oncology, Beatson Oncology Centre, Western Infirmary, Glasgow, G11 6NT, [3] United Kingdom, Institute of Cancer Studies, St.James's University Hospital, Leeds LS9 7TF

Interferon-α (INF) is the standard treatment for patients with metastatic renal cell carcinoma. Renal cell carcinomas are highly vascular with elevated levels of TNF-α, an angiogenic cytokine. Thalidomide (T) is anti-angiogenic and increases the degradation of the mRNA of TNF-α. We report results of an interim analysis of our randomised study to investigate the role of thalidomide in combination with the INF-α.
Metastatic renal cell carcinoma patients were randomised to receive either INF-α alone in the dose of 4.5 MIU SC for the first two doses followed by 9 MIU SC three times a week or in combination with thalidomide 200mg/day orally. Randomisation is based on three prognostic groups, good, moderate or poor.
Patients characteristics were as follows: 34 patients, 22 male, 12 female, median age 57 years (range 44-76), ECOG PS= 0 to 1 in 30 and PS=2 in 4, 9 in good prognostic group, 19 in moderate and 6 in poor prognostic groups.16 received INF-α alone (INF) and 18 received INF-α+Thalidomide (INF+T). 20 completed ≥ 12 weeks of treatment. In the 20 evaluable patients for response the response rates were as follows: in INF arm (12): CR 1, SD 9 and PD 2; in INF+T arm (8): PR 1, SD 2 and PD 5. In 14 who received < 12 weeks treatment, 1 withdrew from study, 13 (5 in INF arm and 8 in INF+T arm) withdrew due to toxicity. GIII toxicities were as follow: anaemia (0 vs.4: INF vs. INF+T), skin rash (0 vs. 2: INF vs. INF+T), dizziness (0 vs.2: INF vs. INF+T), fatigue (5 vs. 2: INF vs. INF+T), anorexia (1 in both arms), thrombocytopenia (1 vs. 0: INF vs. INF+T), neutropenia (2 in both arms). GII peripheral neuropathy was observed more in INF+T arm (0 vs. 3: INF vs. INF+T).
INF-α and thalidomide combination is reasonably well tolerated but with increased incidence of anaemia, skin rash and peripheral neuropathy in the combination arm.

### P36

### IDENTIFICATION OF MARKERS/ANTIGENS IN RENAL CELL CARCINOMA

RA Craven [1], AJ Stanley [1], JS Dods [2], RD Unwin [1], S Hanrahan [2], N Totty [2], S Lloyd [3], P Harnden [4], PJ Selby [1], RE Banks [1]
[1] United Kingdom, Cancer Research UK CLinical Centre, St james's University Hospital, Leeds, [2] United Kingdom, Cancer Research UK Protein Sequencing Laboratory, Lincolns Inn Fields, London, [3] United Kingdom, Department of Urology, St James's University Hospital, Leeds, [4] United Kingdom, Department of Pathology, St James's University Hospital, Leeds

Renal cell carcinoma (RCC) accounts for 2-3% of adult malignancies with a large number of patients having locally advanced or metastatic disease at the time of diagnosis. RCC remains highly refractory to current chemotherapy and radiotherapy regimens, although promising responses to immunotherapy have been reported in a significant subset of patients. Currently there are no reliable markers/antigens for monitoring/detection of disease or for use as therapeutic targets. The aim of this study was to identify such molecules using proteomic-based approaches.
Two dimensional gel electrophoresis in conjunction with mass spectrometry has been used to examine the protein profiles of primary cultures of epithelial cells grown from matched normal kidney cortex and RCC samples. Comparative analysis highlighted 43 proteins upregulated in at least 3 out of 5 RCC primary cell lines and 29 proteins down regulated in at least 4 out of 5 RCC primary cell lines. The identities of 63 of these proteins have been determined to date. Changes in the expression of a number of the proteins identified here have been previously described in RCC, including Hsp27 and several glycolytic and mitochondrial enzymes. In addition several of the proteins found to be differentially expressed in this study are novel findings. Several of these proteins are currently undergoing downstream validation using a panel of primary and established cell lines as well as normal and malignant renal tissue samples.
The results of this study will be presented and compared to proteins identified by a similar proteomic analysis of whole tissue lysates.

### P37

### PREDICTIVE VALUE OF P53 IN DETECTION OF RECURRENCE AND PROGRESSION IN T1 G2-G3 TRANSITIONAL BLADDER CARCINOMA

M Hamdi [1], AG Smith [2], A O'Grady [2], DP Hickey [1], EW Kay [1]
[1] United Kingdom, UROLOGY DEPARTEMENT BEAUMONT HOSPITAL, DUBLIN, [2] Ireland, PATHOLOGY DEPARTEMENT, BEAUMONT HOSPITAL AND THE ROYAL COLLEGE OF SURGEONS IN IRELAND, DUBLIN

**Introduction:** Much controversy surrounds the management of T1 G2-G3 transitional cell carcinoma (TCC) of the bladder. Radical surgery is over treatment in at least 50% of cases. One of the most common genetic changes associated with all types of cancer in humans is an alteration of the p53 gene. Our study helps to determine whether p53 expression in T1 G2-G3 bladder cancer can be correlated with disease recurrence or progression.
**Patients and Methods:** From January 1991 until March 2003, 83 patients were diagnosed with T1 G2-G3 TCC of the bladder. Six (7%) patients were excluded due to insufficient pathological material. Immunohistochemical staining for p53 was carried out on paraffin-embedded tumour specimens using the p53-DO7 murine monoclonal antibody. Immuno-reactivity was categorized as either positive (reactivity in 10% or more tumour cells) or negative.
**Results:** Median follow up was 3.5 years (range 6mts to 12yrs). Fifty-four (70.1%) tumours were p53 positive while 23 (29.9%) tumours were p53 negative. Thirty-seven (68.52%) of the positive cases recurred while 15 (65.2%) of the negative cases recurred (p 0.56). Seventeen (31.48%) of the p53 positive cases progressed while 7 (30.34%) of the negative group progressed (p 0.66).
**Conclusions:** Our results indicate that the percentage of p53 immunomarked cells cannot currently be used to predict recurrence and progression in T1 G2-G3 transitional bladder cancer

### P38

### CODING OF SUPERFICIAL BLADDER CANCER – AUDIT COMPARING A DISTRICT GENERAL HOSPITAL (DGH) AND ITS REGIONAL CANCER INTELLIGENCE UNIT (RCIU)

D Hayne , BK Somani , C Mellors , AM Light , H Ojha , DR Thomas, MC Foster
[1] Sandwell & West Birmingham Hospitals NHS Trust, Birmingham, United Kingdom, [2] Good Hope Hospital, Birmingham, United Kingdom, [3] West Midlands Cancer Intelligence Unit, Birmingham, United Kingdom

Coding of superficial bladder cancer has been controversial. Accuracy of regional cancer registry data has been questioned. Coding of non-invasive TCC bladder from our district general hospital (DGH) was compared with the regional cancer intelligence unit (RCIU).
All cases of superficial non-invasive bladder cancer (pTa and CIS) over a 5 year period 01/01/1996 – 31/12/2001 were identified from DGH database and matched with RCUI registry data using NHS no, hospital no, DOB and patient details. Both databases were further interrogated about individual cases where differences were identified between the data sets. The assigned histopathological codes were also compared.
Of 402 identified cases 389 (97%) were successfully matched with the regional registry. Of the 13 (3%) missing cases 10 were not registered and 3 were registered but only with another primary tumour.
14 (3.5%) were coded as a different urological cancer site (renal pelvis/ ureter not bladder) due to: no definite cancer being identified (DGH coding error –3 patients), no pathology report being sent to /received by RCUI (communication error –2 patients) or the cancer site being miscoded due to a known problem with multiple site coding (RCIU coding policy variation -10 patients).
50 (12%) cases showed discrepancy in the assigned histological code. The majority had been miscoded as non-invasive by DGH probably due to use of an older and potentially misleading coding system. In a few cases no pathology report had been sent/received or the case progressed to invasive disease. No cases were miscoded by RCUI.
RCUI database is acceptably complete and accurate for non-invasive bladder cancers identified from DGH. DGH coding of non-invasive bladder cancers is not as consistent or accurate as RCIU. Nationally agreed coding practices would be beneficial.

© 2004 Cancer Research UK

### P39

### SINGLE CENTRE EXPERIENCE OF OXALIPLATIN (L-OHP) FOR ADVANCED COLORECTAL CARCINOMA (ACC) IN THE WAKE OF NICE

C Thomas , Z Kassam , R Glynne-Jones , M Harrison
Mount Vernon Cancer Centre, London, United Kingdom

INTRODUCTION: 5-year survival of patients with ACC with unresectable liver metastases is 3%. 5-year survival of 20 to 45% has been reported with surgical resection of hepatic metastases. Neoadjuvant chemotherapy may enable liver resection in patients who are initially unresectable and long-term survival is similar to that of a priori surgical candidates. In March 2002 NICE issued guidance that L-OHP in combination with 5 Fluorouracil/folinic acid (5FU/FA) should be considered as first line therapy in ACC with metastases confined to the liver that may become resectable. We present our experience.
METHODS: 41 patients with ACC with liver metastases in whom L-OHP/5FU/FA was planned were prospectively entered into a pharmacy database between 1st March 2002 and 1st February 2004.
RESULTS: Mean age was 60 years (32-78), with M:F of 1.5: 1.Synchronous:metachronous liver metastases was 3:1. 36 (88%) patients have completed chemotherapy to date and been restaged. Of these 15 (42%) had a partial response, 9 (25%) had stable disease, and 12 (33%) progressive disease (using the RECIST criteria). Of these 36 patients 21 (58%) patients were referred for consideration of liver resection. 6 (17%) patients underwent successful liver resection, 1 patient was found to have unresectable disease at laparotomy, 9 were turned down and 5 are awaiting an opinion. Mean follow up from the start of chemotherapy was 14 months (2.0-22). 10 patients (24%) have died, including 1 patient 10 months following hepatic resection, with a mean time to death from the start of chemotherapy of 9.25 months. Median survival has not been reached.
CONCLUSION: In our cohort L-OHP/5FU/FA produced a PR in 42% and a hepatic resection rate of 17%. Chemotherapy to 'downstage' unresectable hepatic metastases has an important role to play in the management of ACC, as surgical resection offers the only chance of cure.

### P40

### POSITRON EMISSION TOMOGRAPHY (PET) IN POTENTIALLY OPERABLE METASTATIC COLORECTAL CANCER PATIENTS: DOES IT ALTER THE DECISION TO OPERATE?

LM Samuel , M Brooks , V Duddalwar , M Koruth
Aberdeen Royal Infirmary, Aberdeen, United Kingdom

Introduction
The 5 year survival for patients with metastatic colorectal cancer (CRC) confined to the liver who have hepatic resection is 40%, suggesting many have occult metastatic disease. PET scans can detect occult metastatic disease, and improve selection for liver surgery, with some studies suggesting a 70% 3-year survival by using PET.
Aims
To prospectively assess the value of PET scanning by looking initially at alterations in treatment decisions when PET scan result revealed.
Methods
All eligible patients, who had no obvious extra-hepatic metastases on abdo-pelvic CT & CXR, had conventional staging (CT angio-portography, and thin slice thoracic CT scan) and a whole body FDG PET scan. PET reported by a radiologist unaware of other staging results. Initial surgical decision taken & recorded without prior knowledge of PET result and then re-assessed with PET result.
Results
First six months results available: 12 eligible patients, of whom 7 have gone on to have a liver resection. Two of these patients had small volume lung metastases detected but this was felt to be potentially resectable at a later date (successfully completed in one patient). Of those not going on to liver resection, one patient with two tiny lesions had a negative PET and has been kept under close review since. Four had extensive disease detected, including two new primaries detected by PET scanning and confirmed at colonoscopy.
Conclusion
PET scanning altered the decision to operate in 40% of patients, sparing them unnecessary liver surgery, and the NHS the costs associated with this type of surgery. The results are in keeping with similar small studies. A health economic analysis is planned.

### P41

### COMPLIANCE TO CHEMOTHERAPY WITH OXALIPLATIN AND 5FLUOROURACIL IN PATIENTS WITH ADVANCED RECTAL CANCER WHO HAVE RECEIVED PRIOR HIGH-DOSE PELVIC RADIOTHERAPY

A Makris [1], R Glynne-Jones [1], D Sebag-Montefiore [2], L Samuel [3], S Myint [4], E Levine [5], A Martin [1], J Dixon [1]
[1] Mount Vernon Hospital, Northwood, United Kingdom, [2] Cookridge Hospital, Leeds, UK, [3] Aberdeen Royal Infirmary, UK, [4] Clatterbridge Hospital, Wirral, UK, [5] Christie Hospital, Manchester, UK

Pelvic radiotherapy (RT) may compromise the compliance and effectiveness of future chemotherapy (CT).
20 patients (15 male, 5 female), mean age 69 yrs (range 51-75) were studied in a prospective, multicenter, phase II study. All patients had recurrent or metastatic diease. All had received previous pelvic RT: 45Gy 25 fx (15), 45 Gy 30 fx (3), 45 Gy 20 fx (2) and 25 Gy 5 fx (1) at a 16 months prior to this study. Most patients had received concurrent chemoradiation (CRT). Patients were treated with a schedule consisting of oxaliplatin 85 mg/m$^2$ on day 1 and 5FU 400 mg/m$^2$ iv bolus and 600 mg/m$^2$ iv 22hour infusion with folinic acid on day 1 and 2. A maximum of eight cycles of CT were administered every two weeks.
A median number of 7 cycles of CT was given (range 3-8), with 132 cycles being given in total. There were 8 episodes of grade III neutropaenia (1 with sepsis). There were 4 episodes of grade III/IV diarrhoea, two of which required hospitalisation, but all resolved. In 18 evaluable patients patients the overall response rate was 33% (3 CR; 3 PR), with 2 patients having SD.
This study shows that the combination of oxaliplatin, 5FU and folinic acid after high-dose pelvic RT is well tolerated and has comparable efficacy to that seen in other metastatic studies and a dose intensity equal to that achieved in adjuvant studies. Based on the results of this study, adjuvant oxaliplatin is being delivered in a randomised phase III study (CHRONICLE) following CRT.

### P42

### MINICHROMOSOMAL MAINTENANCE PROTEINS (MCMS) AS A POTENTIAL NEW TOOL FOR DIAGNOSIS OF PROSTATE CANCER

JL Watkins [1], T Bailey [1], H Clark [2], J O'Donnell [2], AC Woodman [1]
[1] Cranfield Biomedical Centre, Cranfield University, Silsoe, United Kingdom, [2] Department of Biochemistry, Northampton General Hospital NHS Trust, Northampton, United Kingdom

Prostate cancer is now the most prevalent cancer in men accounting for 20% of all new cancer cases and 12% of cancer deaths in the UK. Current diagnostic tests for the disease include measurement of serum PSA (prostate specific antigen) and digital rectal examination (DRE). These techniques may however be problematic, especially since evidence suggests that PSA is not prostate specific and is affected by a variety of factors including age, sexually activity and life style.
This study aimed to investigate specificity of PSA expression by assessing PSA production in a number of prostatic and non-prostatic cell lines *in vitro*, and to consider MCMs as alternative markers for prostate disease. Cell lines derived from eight different tissue types were examined using both immunocytochemistry and western blotting, with antibodies to PSA and MCM-2, -5 and -7.
The results show that PSA is produced by a number of cell lines, including those derived from prostate, colon, bladder and oral epithelium, providing further evidence that PSA is not prostate specific. As an alternative marker, MCMs show differences in the intensity of expression and locality of expression, when cell lines derived from normal prostate and cancerous prostate tissue were examined.
Due to an increasing body of evidence including the results of this study, the measurement of serum PSA may be providing misleading information in the diagnosis of diseases of the prostate. Our study also shows that MCMs may be a novel molecular marker for prostatic cancer, but further work needs to be performed on tissue, to conclude whether MCMs will be a useful diagnostic tool.

© 2004 Cancer Research UK

### P43

### PHOTODYNAMIC THERAPY FOR PROSTATE CANCER – A NOVEL TECHNIQUE FOR ASSESSING LIGHT TRANSMISSION IN THE PROSTATE

CM Moore [1], CA Mosse [1], I Hoh [1], H Payne [2], D Rickards [3], SG Bown [1], ME Emberton [4]
[1] United Kingdom, National Medical Laser Centre, University College London, London, [2] United Kingdom, Myerstein Institute of Oncology, University College London Hospitals Trust, London, [3] United Kingdom, Department of Imaging, University College London Hopsitals Trust, London, [4] United Kingdom, Institute of Urology, University College London, London

Aims
Photodynamic therapy (PDT) uses a light activated drug to cause necrosis, whose depth depends on the penetration depth (PD) of light. Previous studies have measured PD in up to three positions per prostate with wavelengths of up to 665 nm. These wavelengths can be absorbed by haemoglobin, leading to large variation in PD. Newer drugs are activated by light of longer wavelengths. It is predicted that they will have increased PD, and will not be absorbed by haemoglobin. We aimed to use a novel technique to determine PD in multiple positions throughout the prostate, with 763nm light.
Procedures
Patients having high dose rate (HDR) brachytherapy for prostate cancer have multiple transparent plastic needles in the prostate for two days. These are used to carry radioactive iridium wires but are also suitable for carrying optical fibres. Laser light was delivered along the needles using a cylindrically diffusing optical fibre. An isotropic detector was placed sequentially in nearby needles, and optical power at different distances from the source was measured with a light meter. Needle separation was measured on CT.
Major findings
PD is the depth at which 63% of light intensity is lost. It is calculated from the diffusion theory approximation to the Boltzmann transport equation. The mean PD over 600 readings was 5.5mm ( 2.7-12.1mm).
Significance
The PD showed greater variability than expected. As this variation is proportional to the depth aimed to treat, absolute variation will need to be minimised by reducing the needle separation to less than ten mm.
Conclusion
PDT for prostate cancer will require multiple interstitial light sources, similar to the needle configuration used for HDR brachytherapy.

### P44

### PHOTODYNAMIC THERAPY FOR PRIMARY PROSTATE CANCER – A PILOT STUDY USING MTHPC

CM Moore [1], TR Nathan [1], WR Lees [2], A Freeman [3], CA Mosse [1], M Emberton [4], S G Bown [1]
[1] United Kingdom, National Medical Laser Centre, University College London, London, [2] United Kingdom, Department of Imaging, University College London Hospitals Trust, London, [3] United Kingdom, Department of Histology, University College London Hospitals Trust, London, [4] United Kingdom, Institute of Urology, University College London, London

Aims
To determine whether photodynamic therapy (PDT) using meso tatra hydroxy phenyl chlorin (mTHPC) could be a primary treatment for organ confined prostate cancer.
Procedures
Six men with histologically proven prostate cancer, who were unsuitable for or declined radiotherapy, surgery or active surveillance were studied. The mean pre-treatment PSA was 7.7 ng/ml (range 2.7-15), with a Gleason score of 3 + 3 in all patients.
The photosensitiser mTHPC was given intravenously and activated after 2 –5 days by 652nm light delivered transperineally to the prostate using MRI guidance. Areas of cancer were treated under local anaesthetic and sedation according to MRI or biopsy results. The peripheral zone of the same lobe was also treated. 4 out of 6 patients had 2 treatments.
Major findings
Early MRI changes indicated extensive oedema, with patches of ill defined necrosis. This necrosis was well defined at 1 month and was not seen at 3 months. Histology revealed areas of necrosis and fibrosis at 1 month and fibrosis at 2 months. 8 out of 10 treatments resulted in a PSA reduction (mean reduction per patient 4.0 ng/ml, range –2.0 - 10).
2 treatments led to catheterisation for 9 and 19 days respectively. 1 patient had incontinence requiring 1 pad per day for 4 months following a 2nd PDT treatment.
Significance
This is the first report of the use of PDT as a primary treatment for prostate cancer. Necrosis followed by fibrosis, and a PSA reduction were seen.
Conclusion
PDT for primary prostate cancer merits further investigation.

### P45

### CASE ASCERTAINMENT IN PROSTATE CANCER STUDIES-HOW RELIABE ARE HISTOPATHOLOGY DATABASES ALONE?

S Evans [1], C Corbishley [2], G Muir [3], D Gillatt [4], Y Ben-Shlomo [5], R Persad [5]
[1] United Kingdom, Department of Social Medicine, University of Bristol, Bristol, [2] United Kingdom, St.George's Hospital, London, [3] United Kingdom, King's College Hospital, London, [4] United Kingdom, Southmead Hospital, Bristol, [5] United Kingdom, Bristol Royal Infirmary, Bristol

Introduction
Identifying all patients with a specific cancer diagnosis in a hospital is essential to perform accurate clinical audit and for precise registry reporting. The objective of this study was to determine how many cases of prostate cancer would be missed if only histopathology databases were the source of case identification.
Patients and Methods
As part of an ongoing cohort study of men with prostate cancer, all eligible histological and clinically diagnosed cases of prostate cancer were sought at four UK hospitals. Multiple hospital databases were reviewed: Histopathology, Patient Administration System (PAS) and Biochemistry (PSA ≥ 10ng/ml). Case summaries of all patients with a possible or probable clinical diagnosis of prostate cancer were reviewed by a panel of six Consultant Urologists to determine whether there was sufficient evidence for a clinical diagnosis of prostate cancer.
Results
Of a total of 1201 cases identified, 95% had a histological diagnosis, and 5% a clinical diagnosis of prostate cancer. Additional cases (n=147) identified from PAS and PSA database review constituted 12.2% of the total cohort. 89 of these cases were subsequently found to have positive histology not recorded on the histopathology databases. The original histopathology databases would have only identified 88% (95% CI 85.8 to 89.6) of all the cases and 92.2% (95% CI 90.5 to 93.7) of those cases with a histological diagnosis.
Conclusion
Multiple-source case ascertainment ensures that all cases of prostate cancer are identified. Hospital studies relying on a single histopathology database may significantly underestimate the true number of cases managed in their unit. This may also result in a significant under-reporting of prostate cancer to Cancer registries.

### P46

### THE ACUTE URINARY TOXICITY OF COMBINED HIGH DOSE RATE BRACHYTHERAPY BOOST AND EXTERNAL BEAM IRRADIATION FOR HIGH RISK PROSTATE CANCER

NA Anyamene , O Severn , D D'Souza , J Solano , HA Payne
Meyerstein Institute of Oncology, London, United Kingdom

INTRODUCTION-A high proportion of patients who present with prostate cancer in the UK have intermediate or high risk disease. There are encouraging results in the USA and Europe for radiotherapy dose escalation using a combination of external beam radiotherapy (EBRT) with a high dose rate (HDR) brachytherapy boost for this group of patients. We present the acute urinary toxicity for the first 30 patients treated at our centre in London.
METHODS- Entry criteria were any of: stage T3a N0 M0, Gleason grade >8, PSA >20. Patients were initially treated with an HDR boost. Applicator needles were inserted through a perineal template with the aid of transrectal ultrasound. The treatment volume was CT planned and the dose given by an iridium HDR afterloading Microselectron™. The prescribed dose was 16.5Gy in 3 fractions with a minimal interfraction interval of 6 hours. EBRT (46Gy in daily 2Gy fractions) was given to a CT planned volume of prostate and periprostatic tissues.
RESULTS- The age range was between 52 and 79 years (mean = 65years) 70% stage T3a, 76% Gleason grade >8, 40% PSA >20). All had a minimum of 6 months follow up from completion of radiotherapy (range 6-46 months). Acute urinary toxicity was graded using the Mameghan scoring system. Grade 2 urinary toxicity at the end of brachytherapy was nocturia 33%, haematuria 0%, incontinence 7%, dysuria 10% with Grade 1 toxicity nocturia 17%, haematuria 7%, incontinence 3%, dysuria 33%. At the end of EBRT Grade 2 toxicity was nocturia 20%, haematuria 0%, incontinence 3% dysuria 50%, with Grade 1 toxicity nocturia 50%, haematuria 3%, incontinence 7%, dysuria 20%. At 3 and 6 months after completion the urinary morbidity was identical to that measured in the pre-treatment assessment.
CONCLUSION- Brachytherapy boost in combination with EBRT is well tolerated with small risks of Grade 2 toxicity immediately after the procedure. The side effects rapidly reduce with no additional toxicity at 3 and 6 months after completion of all treatment

© 2004 Cancer Research UK

### P47

### ANALYSIS OF NEP AND ECE-1 METALLOPROTEINASE EXPRESSION IN PROSTATE CANCER TISSUE MICROARRAYS

E Leah , AJ Turner , BA Usmani
University of Leeds, Leeds, UK

Prostate Cancer (PC) cells which survive androgen withdrawal therapy can switch allegiance to alternative growth pathways, fuelled by mitogenic peptides, which are normally inactivated by zinc metalloproteinases belonging to a small family of Neprilysin (NEP)-like enzymes. In human PC, NEP loss is recognised as contributing to metastatic androgen-independent PC progression by allowing mitogenic peptides such as endothelin-1 (ET-1) to stimulate cell proliferation. ECE-1, a close homologue of NEP, generates active endothelin (ET-1). We have shown that highly metastatic, androgen-independent PC cell lines express high levels of ECE-1 whereas little ECE-1 is seen in poorly metastatic, androgen-sensitive LNCaP cells. We have also shown a tight inverse correlation between PC expression of NEP and ECE-1. In this study, analysis of archive patient PC tissue microarrays revealed 54% high grade and 19% low grade patient tumours expressed ECE, whereas 15% high grade and 25% low grade tumours expressed NEP. ECE-1 is recognised to exist as four isoforms a,b,c,d. ECE-1c appears to be the common isoform present on cell membranes both in epithellia and stroma of highly malignant tumours. On the other hand, ECE-1a has been unexpectedly recognised in the nucleus of malignant cells and the significance of this remains to be explored. Interestingly, TMA cores representing benign prostatic hyperplasia (BPH) revealed mainly NEP expression (81%).
Conclusion: the data presented here shows an inverse correlation between NEP and ECE-1 in prostate TMA's. This data needs to be correlated with remaining clinical evidence, such as PSA levels, Gleason score, AR status etc, in order to identify a value for ECE and NEP as prognostic or therapeutic markers for prostate cancer.

### P48

### DO PROSTATE CANCER PATIENTS AND THEIR CARERS HAVE A REALISTIC KNOWLEDGE OF THE EPIDEMIOLOGY OF PROSTATE CANCER?

S Evans [1], K Anson [3], G Muir [4], B Patel [1], R Persad [1], Y Ben-Shlomo [1]
[1] United Kingdom, Department of Social Medicine, University of Bristol, Bristol, [2] United Kingdom, Bristol Royal Infirmary, Bristol, [3] United Kingdom, St.George's Hospital, London, [4] United Kingdom, King's College Hospital, London

Introduction
There is little information on the lay epidemiology of prostate cancer. The objective of this cross-sectional survey was to determine knowledge of the epidemiology of prostate cancer amongst men with prostate cancer or their carers.
Patients and Methods
Prostate cancer patients or their carers (in the case of deceased patients) were sent a questionnaire within an ongoing historical cohort study of multi-ethnic communities in the UK. This included several questions on the epidemiology of prostate cancer as well as detailed socio-demographic variables from which a score for socioeconomic status (SES) was derived.
Results
Absolute risk of prostate cancer was over-estimated so that 64% of the sample (n=271) believed that amongst men over 65 years of age annual risk of prostate cancer diagnosis was 10% or more. Higher SES was associated with a greater knowledge of the absolute and relative risk of prostate cancer. Subjects were asked if Black men had a greater, lesser or similar risk of disease compared to White men. 80% of Black men felt that there was no ethnic difference or the risk may even be less for Black men. Risk factors chosen in order of popularity were age, family history and smoking. More affluent subjects favoured family history whilst smoking, despite no convincing epidemiological evidence, was more likely to be chosen by poorer respondents.
Conclusions
The perceived risk of prostate cancer was overestimated. Most Black men, however, did not seem to be aware that they may have been at a greater risk of being diagnosed with prostate cancer. Comparisons with healthy population samples are needed to highlight future educational needs around prostate cancer awareness.

### P49

### ACUTE URINARY MORBIDITY DURING CONFORMAL RADIOTHERAPY FOR PROSTATE CANCER

S Evans [1], R Jones [1], J Graham [2], R Persad [1]
[1] United Kingdom, Bristol Royal Infirmary, Bristol, [2] United Kingdom, Bristol Haematology & Oncology Centre, Bristol

Introduction
Although long-term urinary toxicity from radical three-dimensional conformal radiotherapy (3D-CRT) for localised prostate cancer appears limited, there remains a strong clinical impression of a transient increase in urinary symptoms and deterioration in quality of life (QoL) during treatment. The objective of this study was to determine the immediate effects of 3D-CRT for prostate cancer on lower urinary tract symptoms and QoL.
Patients and Methods
24 consecutive patients with localised prostate cancer were recruited into a prospective study in which the International Prostate Symptom Score (IPSS) and American Urological Association QoL score were serially recorded from baseline, weekly during 3D-CRT, and at four and eight weeks following the end of treatment.
Results
Mean IPSS (0-7mildly symptomatic; 8-19 moderately symptomatic; 20-35 severely symptomatic) rose from 9.75 at baseline to 20.2 by the end of radiotherapy (95% CI for difference 6.3 to 14.6, p<0.001). 50% of patients developed "severe" urinary symptoms by IPSS classification compared to only 10% at baseline. Mean AUA QoL score due to urinary symptoms (0="delighted" to 6="terrible") demonstrated a comparable pattern of deterioration from 2.3 at baseline to 3.9 by the end of treatment (95% CI for difference 0.9 to 2.2, p<0.0001). By eight weeks post-radiotherapy mean IPSS and QoL score had returned to baseline levels.
Conclusions
3D-CRT for localised prostate cancer is associated with a significant acute and transient rise in the IPSS and deterioration in QoL. Further studies to explore the aetiology and possible prevention of these significant symptoms are warranted.

### P50

### EPIGENETIC INACTIVATION OF THE *HSPRY2* GENE IN HUMAN PROSTATE CANCER

AB McKie , DA Douglas , S Olijslagers , JM Graham , MM Omar , CN Robson , HY Leung
Univ. of Newcastle, Newcastle, United Kingdom

Fibroblast growth factors (FGFs) and their receptors are key modulators of normal prostate proliferation with their over-expression associated with aggressive prostate cancer (CaP) and less favourable survival outcome. Recent studies have highlighted the importance of negative regulation of growth factor signalling systems through mechanisms involving the ubiquitin ligase c-Cbl and inhibitory proteins such as Sprouty.
Quantitative RT-PCR was carried out on a cohort of resected CaP and BPH specimens: high grade (poorly differentiated) CaP expressed Spry2 at significantly lower levels than both BPH and well differentiated tumours (p= 0.004). We therefore hypothesise that the observed reduced Spry2 expression is related to the process of prostate carcinogenesis.
The *hSPRY2* gene maps to the long arm of chromosome 13 with CGH and LOH studies implicating genetic loss associated with CaP. The existence of a large CpG island ($OBS_{CpG}/EXP_{CpG}$ ratio of 0.92) led us to investigate the possibility of epigenetic inactivation at this locus. Treatment of LNCaP and PC-3M cells with 5-aza-2'-deoxycytidine (Aza dCR) led to an increase in Spry2 expression from barely detectable levels to moderate levels. Using a combination of methylation specific PCR, and direct sequencing, we have observed extensive hypermethylation of the CpG island in 85% of high grade CaP cases (n=21). Seven control cases of benign prostatic hyperplasia (BPH) were however unmethylated.
Furthermore, selected CaP tumors with reduced Spry2 expression in quantitative RT-PCR were shown to be extensively hypermethylated and control BPH cases with high levels of Spry2 expression were unmethylated. Extensive SSCP analysis yielded little evidence of mutation within the *hSPRY2* gene, however 40-47% LOH was observed with close flanking marker D13S1277 extending distally to D13S266.

© 2004 Cancer Research UK

### P51

**SOLUBLE FGFR (SFGFR) SYNERGISES WITH PACLITAXEL AND GAMMA-IRRADIATION IN SUPPRESSING HUMAN PROSTATE CANCER GROWTH *IN VITRO***

B Gowardhan , C.N. Robson , H.Y. Leung
Prostate Research Group, School of Surgical and Reproductive Sciences, Newcastle University, Newcastle upon Tyne, United Kingdom

The fibroblast growth factor (FGF) family is implicated in prostate cancer, making them important targets for therapeutic agents. sFGFR is the extra-cellular domain of the native FGF receptor (FGFR) which suppresses *in vitro* FGF induced cellular signalling and function. We studied the *in vitro* effects of combining sFGFR gene therapy with paclitaxel and gamma-irradiation.
Proliferation and colony formation of DU145 cells were assessed using recombinant adenoviruses coding for sFGFR1iiib (AdIIIcR1) at a dose of 100 viral particles per cell and various doses of paclitaxel and gamma irradiation.
Proliferation of AdIIIcR1 infected DU145 cells was suppressed by 45% when combined with paclitaxel at 2.5nmol/ml compared to 1% for controls (p=0.005). Similarly, colony formation in AdIIIcR1 infected cells was suppressed by 99% when combined with 1nmol/ml of paclitaxel compared to 40% for controls (p=0.0007). Proliferation of AdIIIcR1 infected cells was suppressed by 45% when combined with gamma irradiation at 2Gy compared to 8% for controls (p=0.0003). Similarly, colony formation in AdIIIcR1 infected cells was suppressed by 50% when combined with 2Gy of gamma irradiation compared to 13% in controls (p=0.0001).
In conclusion, sFGFR synergises with paclitaxel and gamma irradiation in suppressing proliferation and colony formation of DU145 cells. It therefore is a promising agent in the treatment of prostate cancer which may help reduce the dose of chemo- and radio- therapy without compromising on attaining the desired therapeutic effect.

### P52

**PROSTATE CANCER – HORMONES FIRST, RADIOTHERAPY LATER, OR LATER STILL**

KJ Turner , C Parsons , SA McNeill , D McLaren
Western General Hospital, Edinburgh, United Kingdom

Introduction and Aims: Contemporary treatment for locally advanced prostate cancer (PC) includes external beam radiotherapy (EBRT) with concomitant androgen ablation. We have a cohort of patients who were treated with prolonged androgen ablation prior to radical EBRT. The outcome of such an approach has not been evaluated previously.
Patients and Methods: Patients with PC who had been treated with androgen ablation for at least 10 months prior to radical EBRT were identified from our EBRT database. Baseline tumour characteristics were recorded and outcome variables including time to progression and time to death following EBRT were obtained from notes review. Data were analysed by Cox regression analysis.
Results: 45 eligible patients (mean age 67 years) were diagnosed between 1969 and 1999. Mean follow up was in excess of 7 years (86 months (range 36-192) months. Mean duration of androgen ablation prior to EBRT was 40 months (range 10-84 months). Overall 5-year progression free survival was 36%. PSA nadir on androgen ablation prior to EBRT, and PSA nadir post EBRT were both significantly associated with prolonged progression free survival (p=0.03 and p=0.01 respectively). PSA at diagnosis was not a significant predictor of outcome. A shorter duration of androgen ablation prior to EBRT was associated with significantly prolonged progression free and overall survival (p=0.03 and p=0.01 respectively).
Conclusions: The optimal sequence, timing, and duration of androgen ablation in patients with PC undergoing EBRT with curative intent remain undetermined. Results presented here compare favourably with results for early EBRT and androgen ablation in high risk PC. A shorter period of androgen ablation and good biochemical response to androgen ablation and EBRT are associated with better outcome. A prolonged period of hormone ablation should not necessarily exclude PC patients from receiving potentially curative radical EBRT.

### P53

**ISSUES OF DOSIMETRIC ANALYSIS OF BRACHYTHERAPY SEED IMPLANTS**

D Power , I Coles , K Wood , S Stewart
United Kingdom, Charing Cross Hospital, London

Introduction: Prostate brachytherapy in early prostate carcinoma in appropriately selected patients achieves comparable survival and local control rates to radical surgery/radiotherapy. The dose delivered to 90% of the prostate gland (D90) correlates with clinical outcome.
Methods: 71 patients were treated with palladium-103 seed implant between January 1999 and 2003. Implant quality audited using American Brachytherapy Guidelines (1999). Post implant dosimetry using Computerised Tomographic imaging was calculated using MMS Planning System (Variseed). Rectal dose volume histograms (RDSH) and urethral doses also assessed.
Results: Average age 61 years (range: 48 - 73). Average number of Pd-103 seeds implanted = 77 (55-106). Pre-treatment ultrasound assessed prostate volumes: 82% $\leq$ 40cm$^3$, 0% $\geq$ 60cm$^3$. Significant differences observed in the pre implant ultrasound & post implant CT defined prostate V100 & V200. Ratio of Ultrasound prostate volume: CT prostate volumes = 1.45. D90 adequate in 60% of patients analysed.
Discussion: Difficulty outlining post-implant prostate gland using CT imaging is well recognised. Implant quality assessment is dependent on accurate prostate volume definition. This is difficult to define with the use of differing imaging modalities pre and post implant. No clear correlation between rectal dose surface histogram and rectal toxicity. Assessment of rectal dose practically difficult. We also identified problems localising urethra using current imaging techniques in the assessment of urethral dose.
Conclusions: Variations in imaging modalities pre and post implants results in difficulties in dosimetric analysis, reflected in the V100 & D90 values. Important to audit adequacy of prostate brachytherapy implant technique. Rectal and urethral dose assessment difficult with current imaging techniques. There are currently no clearly defined national standards to audit against.

### P54

**WEIGHT TRAINING HAS BENEFICIAL PHYSICAL AND PSYCHOLOGICAL EFFECTS IN PROSTATE CANCER (PC) PATIENTS ON ANDROGEN DEPRIVATION THERAPY (ADT)**

NSA Stuart [1], SM Marcora [1], N Callow [1], SJ Oliver [1], W Saxton [2]
[1] United Kingdom, University of Bangor, Bangor, [2] United Kingdom, Gwynedd Hospital, Bangor

**Background**: ADT is the standard treatment for metastatic prostate cancer. However it also causes muscle wasting, fatness, bone loss, fatigue, and reduces physical activity and quality of life (QoL). The aim of this pilot study was to assess the feasibility and effectiveness of progressive resistance training (PRT, "weight training") in reducing or reversing these effects. **Methods**: 10 PC patients (pts) on ADT were randomly allocated either to supervised PRT in the University gym 3 times/week for 12 weeks (training group TG, n=5) or standard treatment alone (control group CG, n=5). Pts were assessed before randomisation and after 12 wks. Physical measures were body composition (measured by DEXA scan), Senior Fitness Test and pedometry. Psychological measures were Bidimensional Fatigue Scale, FACT-Prostate QoL scale and HAD scale. **Results (physical):** One TG subject was dropped from the study for safety reasons (diagnosis of bone metastases shortly after enrolment). The other 4 pts completed 85% of the training sessions. There was a increase in total muscle mass in the TG (2.2 ±1.1 kg) compared to the CG (-0.1 ±0.7 kg, p = 0.02). This was particularly evident in the arms and legs. These changes were not solely due to an increase in extra-cellular water but also intracellular water and protein mass. There were also improvements (p = 0.02) in upper body strength and lower body flexibility as well as non-significant trends to improvements in physical activity, agility and 6-minute walking distance. **Results (psychological):** In the TG compared with the CG there were statistically non-significant trends to improvement in FACT-P (+5.3 points vs –10.4), BFS and HADS. **Conclusion:** PRT is an effective and well tolerated treatment for the adverse physical effects of ADT. It may also reduce the adverse psychological effects. An application has been made to CR(UK) to fund a larger, randomised, controlled study to confirm these results. A work out in the gym may become an important part of the palliative care of PC pts.

© 2004 Cancer Research UK

### P55

### QUANTITATIVE PROTEOMIC ANALYSIS OF ANDROGEN & ANTI-ANDROGEN RESPONSES IN THE LNCAP PROSTATE CANCER CELL LINE

JG Rowland [1], JW Simon [2], AR Slabas [2], CN Robson [1], HY Leung [1]
[1] University of Newcastle upon Tyne, Newcastle upon Tyne, United Kingdom, [2] University of Durham, Durham, United Kingdom

**Introduction.** Proteins responsive to androgen and anti-androgen may be involved in the development of prostate cancer and the ultimate failure of androgen-ablation therapy. These proteins are potential targets for the development of novel therapeutic agents. We have investigated the effect of androgen and anti-androgen stimulation on the androgen responsive prostate cancer LNCaP cell line using 2-dimensional differential in-gel electrophoresis technology (2-DIGE), with the aim of discovering proteins involved in the androgen receptor function in LNCaP cells.

**Methods.** Primed LNCaP cells were stimulated with 10nM R1881, 1μM bicalutamide or vehicle (ethanol) for 72hrs. Cells were harvested and lysed in IEF buffer (9M Urea, 2M Thiourea, 4% CHAPS). Three biological repeats were generated and analysed using 2D-DIGE technology (Amersham Biosciences). Samples were labelled with CyDye fluors, multiplexed and subjected to 2-dimensional electrophoresis (pI range 3-10, linear). Gels were imaged using a Typhoon laser scanner and analysed in the DeCyder software package (Amersham Biosciences). Differential expression was determined by student's t-test and >1.5-fold change in abundance.

**Results.** Gels containing around 3000 protein features per gel were produced, with 77 protein spots showing significant differential regulation, including proteins involved in protein synthesis, antioxidant defence and various signalling pathways. Many have previously been reported to be androgen-responsive or potentially involved in the development and progression of malignancies including prostate cancer.

**Conclusion.** Using 2D-DIGE we have quantitatively identified numerous candidate proteins involved in the cellular response to androgens and anti-androgens. These proteins may be targets for the development of novel therapeutic intervention strategies.

### P56

### ANORECTAL DYSFUNCTION AFTER RADIOTHERAPY FOR UROLOGICAL MALIGNANCY

D Hayne , BK Somani , RS Kushwaha , E Wrightham ,
H Payne , PB Boulos
[1] City Hospital, Birmingham, United Kingdom, [2] The Middlesex Hospital, London, United Kingdom, [3] Department of Surgery, Royal Free and University College Medical School, London, United Kingdom, [4] The Meyerstein Institute, London, United Kingdom

The adverse effects of pelvic radiotherapy on anorectal function may be underestimated. This prospective study aimed to identify symptoms 31 men (median age 70 years) with carcinoma of the prostate (28) and bladder (3) completed incontinence/proctitis symptom scores and anorectal physiological studies before and 6 weeks after pelvic radiotherapy. At 6 months 19 of these patients were studied. Results were compared using the Wilcoxon signed rank test.

Incontinence scores increased: 0 (0-5) v 4 (0-11) (p<0.001) v 3 (0-14) (p<0.007), pre v 6 weeks post- v 6 months post-radiotherapy, median (range) and proctitis scores increased: 0 (0-4) v 2 (0-7) (p<0.001) v 2 (0-5) (p<0.004), pre v 6 weeks post- v 6 months post-radiotherapy, median (range).

Anal canal resting pressure decreased: 83 (35-137) v 79 (26-152) (p<0.136) v 73 (37-97) (p<0.001), pre v 6 weeks post- v 6 months post-radiotherapy, median cm $H_2O$ (range), squeeze pressure was unchanged but maximum tolerated rectal volumes decreased: 245 (115-450) v 194 (112-344) (p<0.005) v 194 (109-350) (p<0.005), pre v 6 weeks post- v 6 months post-radiotherapy, median cm mls (range).

Significant acute symptomatic and manometric anorectal disturbance is seen after RT for prostate and bladder cancer. At six weeks this appears to be related to diminished maximum rectal tolerated volumes. Symptoms at 6 months may be explained by the decrease in anal canal resting pressure together with diminished maximum rectal tolerated volumes.

### P57

### ACUTE RECTAL TOXICITY AFTER CONFORMAL RADIOTHERAPY FOR PROSTATE CANCER. COULD $V_{50}$ OR AUC OF A DVH BE PREDICTIVE FACTORS

NA Anyamene , O Severn , R Subramaniam , HA Payne
Meyerstein Institute of Oncology, London, United Kingdom

**Purpose & Objectives**: The volume of the rectum (as defined by the DVH) receiving the prescribed radiation dose is a well described predictive (and dose limiting) factor of late toxicity following treatment for prostate cancer. There is, however, little literature on predictive factors for acute rectal symptoms. We aim to identify whether there is a relationship between AUC (area under curve) of the rectal DVH (dose volume histogram) or $V_{50}$ (volume of rectum receiving 50Gy) and acute rectal toxicity after CT conformally planned radiotherapy for prostate cancer.

**Methods**: We prospectively analysed 20 consecutive patients with $T_1$-$T_3$ adenocarcinoma of the prostate treated between August –September 2003. Median age 62 years (range 49-77). They received CT conformally planned external beam radiotherapy to an IRCU dose of 74Gy. The rectum was outlined from 1cm above and 1cm below the GTV (gross tumour volume) and included the outer rectal wall. The $V_{50}$ and AUC were identified from the rectal DVH.

Acute anorectal symptoms were assessed at the end of treatment and one month post completion using a 0-11 score questionnaire devised by Talley et al 1997. We looked for a correlation between symptom scores at these times and the $V_{50}$ and AUC.

**Results:** The mean anorectal toxicity score at the end of treatment was 3.68 (range 0-7) and at one month post completion of treatment was 0.52 (range 0-2). The mean $V_{50}$ was 42.31% (range 13.3-70%) and the mean AUC 6552 (range 5999-8207). There was some correlation between rectal symptoms and AUC but no correlation with $V_{50.}$

**Conclusions:** From this study we can conclude that neither AUC nor $V_{50}$ are accurate predictors of acute rectal toxicity following radical radiotherapy to the prostate, but that the AUC of the DVH shows some correlation. We plan to monitor these patients for late sequelae to assess whether AUC is a superior predictive factor than $V_{50}$ for late rectal morbidity

### P57:1

### LOW DOSE CONTINUOUS HYPERFRACTIONATED ACCELERATED RADIATION THERAPY (CHART) AS PREOPERATIVE TREATMENT FOR RECTAL CARCINOMA

S.M Short , A. Makris , R. Glynne Jones , R. Novell , M. Harrison
Mt Vernon Hospital, London, United Kingdom

**Introduction:** To determine the feasibility of low dose CHART as preoperative short-course treatment for locally advanced rectal tumours.

**Methods:** 18 patients with locally advanced rectal carcinoma, between 1998 and 2004, entered a nonrandomised trial of preoperative low dose CHART. Patients received 25 Gy in 15 fractions over 5 days to a standard pelvic field. Toxicity was graded using the RTOG/EORTC acute and late toxicity scoring system. The average follow-up time is 20 months (range 0.7-52 months).

**Results:** 17 patients completed planned radiotherapy treatment and went forward for surgery. One patient discontinued radiotherapy after 17 Gy and did not proceed to surgery. One patient was inoperable at the time of surgery due to local disease extent.

Survival: Overall 4 year survival is 76% and cause specific survival is 87%. 2 patients died in the perioperative period due to sepsis unrelated to tumour or radiotherapy. 2 patients died from metastatic disease, both with primary tumour in-situ. The remainder (14) are alive without evidence of disease

Toxicity: No patients experienced grade 3 or 4 acute toxicity. 1 patient developed grade 2 bowel toxicity and discontinued treatment. There was 1 grade 1 bowel toxicity and 1 grade 1 wound infection after surgery. No patients experienced grade 2, 3 or 4 late toxicity. 5 patients experienced grade 1 bowel toxicity and 5 late urinary symptoms.

**Conclusion:** Low dose CHART as preoperative short-course treatment for rectal carcinoma is feasible with a tolerable side effect profile. These findings compare favourably to series using 25 Gy in 5 daily fractions.

© 2004 Cancer Research UK

### P58

### A RETROSPECTIVE STUDY OF THE MANAGEMENT AND OUTCOME OF PATIENTS WITH STAGE 1 (MARKER NEGATIVE) TERATOMA

RA Herbertson , MQ Hatton , J Coleman , RE Coleman
Weston Park Hospital, Sheffield, United Kingdom

**Purpose**; To identify all patients with stage 1, marker negative teratoma treated within a 5 year period at Weston Park Hospital in Sheffield. To document patient and tumour characteristics, management at diagnosis and relapse, method of detection of relapse and overall outcome of these patients.
**Patients and Methods**; All the relevant information was obtained from the medical notes of the 81 patients identified as having been diagnosed with stage 1 teratoma between 1/1/96 and 1/1/01.
**Results**; 43.2% of patients had a raised tumour markers pre-operatively. 46.9% of patients had tumours which demonstrated at least one histological poor prognosis factor. (46.9% had a major component of undifferentiated structures, 40.7% demonstrated absence of yolk sac tumour and 18.5% had vascular invasion). All 18.5% of patients with vascular invasion received immediate adjuvant chemotherapy (2-3 cycles of BEP). The remaining 64 patients were followed according to the hospital's surveillance program. 20% of patients relapsed. None of the 15 "high risk" patients who received immediate chemotherapy relapsed. 81.3% of those who relapsed had a large component of undifferentiated structures compared to 30.0% in those on surveillance who did not relapse. The commonest method of detecting relapse was routine CT scan. Median time to relapse was 4.8 months. All 16 relapsed patients received chemotherapy. Complete remission was achieved for all patients, and overall survival was 100%. Median disease free survival was 45.2 months.
**Discussion;** The relapse rate at our centre is similar to those documented in the literature. In this study, 2 cycles of adjuvant BEP in patients whose tumour demonstrated vascular invasion seemed to prevent recurrence and was well tolerated. Relapse was detected quickly (usually by CT scan) and following chemotherapy, all patient achieved a complete remission and remain disease free.

### P59

### QUANTITATIVE ANALYSIS OF CYCLIN DEPENDENT KINASE INHIBITORS, P16$^{INK4A}$ & P21$^{WAF1}$, PREDICTS NEOPLASTIC PROGRESSION OF CERVICAL GLANDULAR ABNORMALITIES

A El-Ghobashy , A Shaaban , J Innes , W Prime , CS Herrington
United Kingdom, Liverpool University, Liverpool

El-Ghobashy AA, Shaaban AM, Innes J, Prime W, Herrington CS
Department of Cellular and Molecular Pathology, the University of Liverpool, UK
Introduction: Cyclin-dependent kinase (CDK) inhibitors are important negative regulators of cell cycle progression. Inactivation or downregulation of the cell-cycle inhibitors, p16 and p21, are involved in the carcinogenesis of various human tumors. Less is known about the role of these CDK inhibitors in cervical glandular carcinogenesis.
**Aim:** To investigate the role of CDKs in cervical glandular neoplasia by comparing their expression in lesions of different histological type.
Materials and Methods: Paraffin wax-embedded sections of normal cervix (group 1, n= 11), endometriosis/ tubo-endometrioid metaplasia (TEM) (group 2, n= 19), cervical glandular intraepithelial neoplasia (CGIN) (group 3, n= 33) and invasive adenocarcinoma (group 4, n= 28) were stained using monoclonal antibodies for p16$^{INK4}$ and p21$^{WAF1}$. A quantitative assessment was employed for the analysis of percentage expression of each marker.
**Results:** Increase expression of p16 occurred from normal cervix (median%: 0, IQ 0-1), through endometriosis/TEM (median%: 35, IQ 8-45) and CGIN (median%: 95, IQ 75-100) with no further increase in invasive adenocarcinoma (median%: 90, IQ 80-95). The expression of p21 in the four different groups was [0, IQ 0-0]; [0, IQ 0-3]; [1.5, IQ 0-5] and [9, IQ 2.25-15] respectively. A cut-off value of 50% for p16 expression and 0% for p21 expression identified benign (group 1&2) from neoplastic (group 3&4) lesions with sensitivity of 90% and specificity of 87% for the former and 73% & 76% for the latter.
**Conclusion:** Our data highlight a progressive increase in p16 and p21 expression across spectrum of cervical glandular neoplasia. p16 and p21 are useful markers to differentiate benign (groups 1 & 2) from neoplastic (groups 2 & 3) endocervical lesions.

### P59:1

### DELAYS IN THE DIAGNOSIS OF COLORECTAL CANCER: IN-DEPTH, PATIENT-CENTRED QUALITATIVE STUDY

E.M. Wood [1], R.D. Neal [2], P.L. Heywood [1], K.M. Atkin [1]
[1] United Kingdom, Centre for Research in Primary Care University of Leeds, Leeds, [2] United Kingdom, University of Wales College of Medicine, Cardiff

This study aims to provide a rich and detailed description of the journey from the pre-symptomatic phase through to diagnosis, in order to identify and explain diagnostic delays (patient, primary care and secondary care delays) in colorectal cancer, and to suggest strategies for addressing delays.

The experience of thirty patients, newly diagnosed with colorectal cancer was explored through in-depth semi-structured interviews. The related experiences of a family member and GP was also sought. By integrating the perspectives of the patient, family member and GP using qualitative analysis, key factors were identified that are important in causing diagnostic delays.
Initial primary observations show: Most patients had heard of colorectal cancer before their diagnosis; however the majority of patients were not aware of symptoms specific to colorectal cancer, or of lifestyle/dietary factors that may contribute to/prevent colorectal cancer. The majority did not expect their symptoms to be due to a cancer. Patient responses to their symptoms varied from seeking immediate medical help to a delay in presentation. Rectal bleeding caused more concern and prompted patients to seek medical help more quickly than with abdominal pain or change in bowel habit. Delays occurred despite cancer concerns. Rectal bleeding prompted immediate referral by GPs to secondary care; delays occurred in cases of pain and change in bowel habit. Secondary care delays were caused by long waiting lists for investigations and administration errors.
The results will enable us to appreciate current patient understanding of colorectal cancer and target patient education accordingly; enable clinicians to understand more about patient experiences and perceptions of these experiences; enable us to realise what issues are important, enabling policy and practice to be sensitive to these views which in turn should reduce delays and improve outcomes for these patients.

### P60

### AGE AT PRESENTATION AFFECTS FREQUENCY OF B-RAF MUTATION IN PAPILLARY THYROID CARCINOMA(PTC)

SJ Jeremiah [1], NG Powell [1], LA Stephens [1], JA Bethel [1], TI Bogdanova [2], MD Tronko [2], GA Thomas [2]
[1] Human Cancer Studies Group, Swansea University, Swansea, United Kingdom, [2] Institute of Endocrinology and Metabolism, Kiev, Ukraine

We have investigated the frequency of B-raf mutation in a series of 31 cases of PTC in patients from Ukraine aged between 30 and 61 and 37 cases of PTC from Ukrainian patients aged under 16 at operation, 8 of whom had been born after 1.12.86, and therefore regarded as not exposed to radioiodine from the Chernobyl accident.
Areas of PTC were microdissected from paraffin embedded sections, DNA extracted and the presence of the common V600E B-raf mutation demonstrated by PCR and restriction enzyme digestion. PCR products were sequenced using an ABI sequencer to confirm the presence of the mutation. No mutation in B-raf was observed in any of the paired samples of normal thyroid tissue examined; mutations were confined to the tumour. 18 (58%) of the adult cases harboured a B-raf V600E mutation. Mutation in B-raf was more commonly associated with a classic morphology (12/16; 75%) than a follicular variant (6/12; 50%). The three solid variants of PTC (normally associated with ret rearrangement) in our adult series were negative for B-raf mutation. The overall frequency of rearrangement is similar to that observed in a number of other adult series.
Only 1 of the 37 (2.7%) cases of PTC aged under 18 at operation were positive for B-raf mutation. The positive case was a follicular variant of PTC and was negative for ret rearrangement. Of the 8 PTCs from patients born after 1.12.86, none was positive for B-raf mutation. These results confirm that B-raf mutation is more commonly associated with PTC diagnosed in adult life, and that the Ukrainian adult population shows a similar frequency of B-raf involvement to that observed in adult series from other countries.
The low frequency of B-raf involvement in juvenile PTC is therefore likely to be associated with the age of the patient at diagnosis and morphological subtype of PTC, rather than a radiation aetiology.

© 2004 Cancer Research UK 

